# (2-Amino-7-methyl-4-oxido­pteridine-6-carboxyl­ato-κ^3^ *O* ^4^,*N* ^5^,*O* ^6^)aqua(1,10-phen­an­thro­line-κ^2^ *N*,*N*′)cobalt(II) trihydrate

**DOI:** 10.1107/S1600536812051185

**Published:** 2012-12-22

**Authors:** Siddhartha S. Baisya, Samir Sen, Parag S. Roy

**Affiliations:** a Department of Chemistry, University of North Bengal, Siliguri 734 013, India

## Abstract

In the title compound, [Co(C_8_H_5_N_5_O_3_)(C_12_H_8_N_2_)(H_2_O)]·3H_2_O, a tridentate 2-amino-7-methyl-4-oxidopteridine-6-carboxyl­ate ligand, a bidentate ancillary 1,10-phenanthroline (phen) ligand and a water mol­ecule complete a distorted octa­hedral geometry around the Co^II^ atom. The pterin ligand forms two chelate rings. The phen and pterin ring systems are nearly perpendicular [dihedral angle = 85.15 (8)°]. N—H⋯O, O—H⋯N and O—H⋯O hydrogen bonds link the complex mol­ecules and lattice water mol­ecules into a layer parallel to (001). π–π stacking contacts (involving phen–phen and pteridine–pteridine) are also observed [centroid–centroid distances = 3.670 (2), 3.547 (2), 3.698 (2) and 3.349 (2) Å].

## Related literature

For background to the chemistry of pterins in metalloenzymes, see: Basu & Burgmayer (2011 ▶); Burgmayer (1998 ▶); Fitzpatrick (2003 ▶); Fukuzumi & Kojima (2008 ▶). For structures of related cobalt complexes, see: Acuña-Cueva *et al.* (2003 ▶); Beddoes *et al.* (1997 ▶); Burgmayer & Stiefel (1988 ▶); Funahashi *et al.* (1997 ▶). For structures of related copper complexes, see: Odani *et al.* (1992 ▶). For the electron-shuffling ability of the pterin unit as well as its donor groups and the effect on the geometric parameters of related complexes, see: Beddoes *et al.* (1993 ▶); Kohzuma *et al.* (1988 ▶); Russell *et al.* (1992 ▶). For the synthesis of the pterin ligand, see: Wittle *et al.* (1947 ▶).
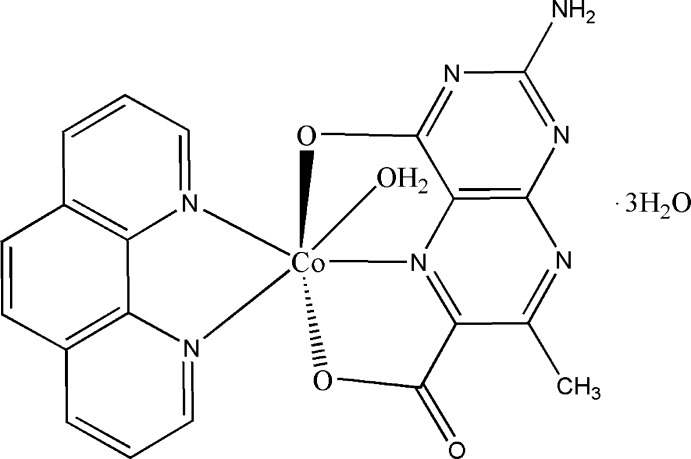


## Experimental

### 

#### Crystal data

[Co(C_8_H_5_N_5_O_3_)(C_12_H_8_N_2_)(H_2_O)]·3H_2_O
*M* *_r_* = 530.36
Triclinic, 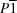

*a* = 8.454 (2) Å
*b* = 9.934 (3) Å
*c* = 13.778 (4) Å
α = 97.534 (4)°
β = 95.281 (4)°
γ = 110.603 (4)°
*V* = 1061.8 (5) Å^3^
*Z* = 2
Mo *K*α radiation
μ = 0.87 mm^−1^
*T* = 110 K
0.23 × 0.11 × 0.04 mm

#### Data collection

Bruker Kappa APEXII CCD diffractometer
Absorption correction: multi-scan (*SADABS*; Sheldrick, 1996 ▶) *T*_min_ = 0.82, *T*_max_ = 0.97
8945 measured reflections
4726 independent reflections
4360 reflections with *I* > 2σ(*I*)
*R*_int_ = 0.030

#### Refinement

*R*[*F* ^2^ > 2σ(*F* ^2^)] = 0.057
*wR*(*F* ^2^) = 0.129
*S* = 1.03
4726 reflections
316 parameters
H-atom parameters constrained
Δρ_max_ = 0.99 e Å^−3^
Δρ_min_ = −0.88 e Å^−3^

### 

Data collection: *APEX2* (Bruker, 2007 ▶); cell refinement: *SAINT* (Bruker, 2007 ▶); data reduction: *SAINT*; program(s) used to solve structure: *SHELXS97* (Sheldrick, 2008 ▶); program(s) used to refine structure: *CRYSTALS* (Betteridge *et al.*, 2003 ▶); molecular graphics: *CAMERON* (Watkin *et al.*, 1996 ▶); software used to prepare material for publication: *CRYSTALS*.

## Supplementary Material

Crystal structure: contains datablock(s) global, I. DOI: 10.1107/S1600536812051185/hy2609sup1.cif

Structure factors: contains datablock(s) I. DOI: 10.1107/S1600536812051185/hy2609Isup2.hkl

Additional supplementary materials:  crystallographic information; 3D view; checkCIF report

## Figures and Tables

**Table 1:** Hydrogen-bond geometry (Å, °)

*D*—H⋯*A*	*D*—H	H⋯*A*	*D*⋯*A*	*D*—H⋯*A*
N7—H141⋯O2^i^	0.85	2.12	2.942 (4)	163
N7—H142⋯O6^ii^	0.84	2.15	2.970 (4)	165
O4—H181⋯O6	0.81	1.93	2.717 (3)	164
O4—H182⋯N5^ii^	0.80	2.25	3.051 (4)	176
O5—H341⋯O1	0.82	2.34	3.079 (4)	151
O5—H341⋯O2	0.82	2.23	2.896 (4)	139
O5—H342⋯N4^iii^	0.82	2.04	2.844 (4)	166
O6—H351⋯O5	0.83	1.92	2.740 (4)	174
O6—H352⋯N5^iv^	0.82	2.05	2.871 (4)	176
O7—H331⋯O5^i^	0.80	2.25	2.941 (4)	145
O7—H332⋯O3	0.81	2.23	2.962 (5)	151

Symmetry codes: (i) 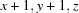; (ii) 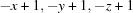; (iii) 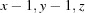; (iv) 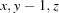.

**Fig. 1.**
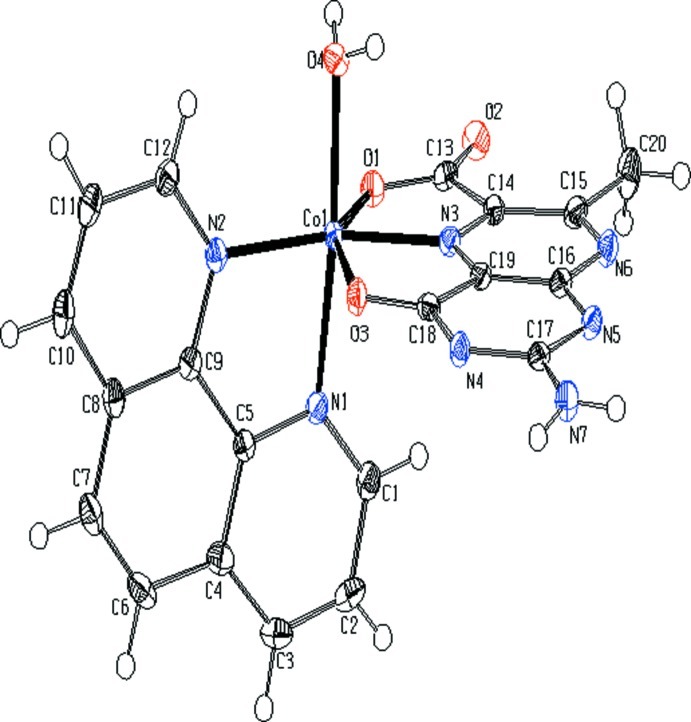
The molecular structure of the title compound. Displacement ellipsoids are drawn at the 50% probability level. Lattice water molecules are omitted for clarity.

**Fig. 2.**
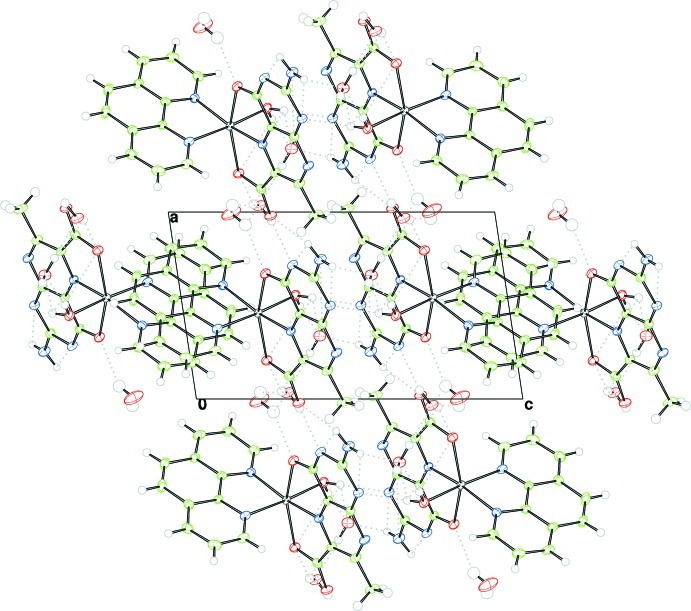
The crystal packing diagram of the title compound, viewed along the b axis. Dotted lines indicate hydrogen bonds, assisting the formation of a layer structure parallel to (001).

**Fig. 3.**
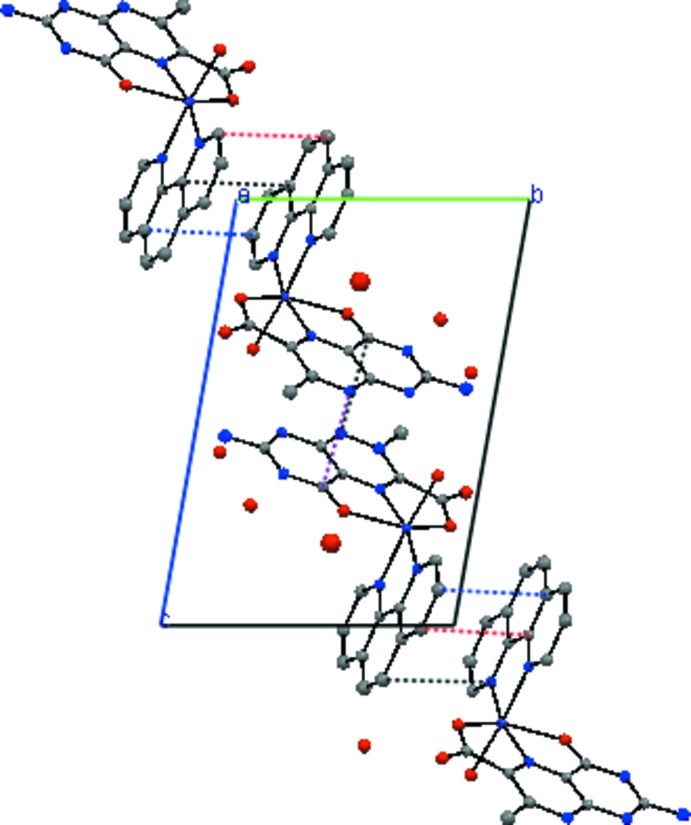
A molecular packing diagram highlighting π–π stacking interactions between two neighbouring phen–phen and pterin–pterin rings, respectively.

## supplementary crystallographic information

### Comment

The primary motivation for pursuing coordination chemistry of pterins is the ubiquitous presence of this heterocyclic system in nature including a substantial number of metalloenzymes (Basu & Burgmayer, 2011; Burgmayer, 1998; Fitzpatrick, 2003; Fukuzumi & Kojima, 2008). Literature survey reveals the existence of only a few X-ray structurally characterized cobalt-pterin/pteridine/lumazine complexes as well as one containing an organocobalt moiety (Acuña-Cueva *et al.*, 2003; Beddoes *et al.*, 1997; Burgmayer & Stiefel, 1988; Funahashi *et al.*, 1997). The concerned ligands usually act as bidentate O,N-donors and none of the above complexes possesses a typical π-acceptor ancillary ligand like 1,10-phenanthroline (phen). In this crystallographic study on the title cobalt(II) complex, possessing both a tridentate pterin ligand and a π-acidic ligand like phen, different aspects are considered, *e.g.* crystal, molecular and electronic structures.

In the title compound (Fig. 1), the stereochemistry around the Co^II^ atom is essentially distorted octahedral with two N atoms of phen, a pyrazine ring N atom (N3) of the pterin ligand and an aqua O atom forming the equatorial plane; two pterin O atoms (O1 and O3) define the longer axial positions, with the phenolate O3 forming the longest axial bond [2.270 (2) Å]. Extent of distortion of this coordination octahedron is much more pronounced as compared to that of the Co(II)-pteridine complexes reported earlier (Acuña-Cueva *et al.*, 2003; Burgmayer & Stiefel, 1988; Funahashi *et al.*, 1997). A major cause of this departure from regular geometry is that the pterin ligand forms two five-membered chelate rings having small bite angles [75.10 (10) and 76.26 (9)°], instead of only one per pteridine ligand for the earlier cases. Location of the short Co1—N3 bond [2.016 (3) Å] in the equatorial plane is consistent with the literature, which suggests a strong cobalt-pterin interaction (Odani *et al.*, 1992). The pterin ligand is coordinated here as a binegative tridentate ONO donor, as evident from the charge balance of this complex. The phen and pterin rings are nearly perpendicular to each other for minimizing the steric repulsion. The Co1—N1 [2.079 (3) Å] and Co1—N2 [2.123 (3) Å] bond lengths are at par with that of the Co1—N3 bond [2.016 (3) Å] and indicate receipt of π-back donation to both phen and pterin rings from the Co(II) centre (d^7^) through dπ–pπ interactions. This process is further strengthened by the presence of π-donating phenolate and carboxylate O atoms around the metal centre (Kohzuma *et al.*, 1988).

For rationalizing the near double bond nature of the O3—C18 [1.265 (4) Å] bond, a hypothesis of Joule (Beddoes *et al.*, 1993; Russell *et al.*, 1992) may be invoked, which suggests withdrawl of electron density from the pyrazine ring N6 by the pyrimidine ring C18-carbonyl group through mesomeric interaction. Formation of the O3—Co1 bond accentuates this electron withdrawal towards O3. The electron-rich N7—C17 [1.337 (4) Å] bond may also participate in this electron transfer. The pyrimidine ring is fairly planer and deviations of the C16/N5/C17 and C17/N4/C18 segments with respect to the N7—C17 multiple bonds are 2.6 and 0.7°, respectively.

In the crystal, intermolecular N—H···O, O—H···N and O—H···O hydrogen bonds (Table 1) link the complex molecules and lattice water molecules into a layer parallel to (001) (Fig. 2). The lattice water molecules are decisive for the crystal packing. Fig. 3 reveals π–π stacking interactions involving two parallel, inversion-related pterin rings within the same unit cell and showing face-to-face distance of 3.283 (4) and 3.366 (4) Å. Again the phen rings display two types of π–π stacking on either side of the unit cell. In one case, the adjacent phen rings are essentially parallel to each other with an average interplanar distance of 3.496 (4) Å; on the other side of the unit cell, the face-to-face seperations between parallel phen rings are 3.578 (4) and 3.629 (5) Å.

### Experimental

2-Amino-4-hydroxy-7-methylpteridine-6-carboxylic acid sesquihydrate (C_8_H_7_N_5_O_3_.1.5H_2_O) was obtained by published procedure (Wittle *et al.*, 1947). The title complex was prepared by the dropwise addition of an aqueous alkaline solution (NaOH: 11 mg, 0.275 mmol) of the pterin ligand (31 mg, 0.125 mmol) to a warm (311 K) aqueous reaction medium containing CoSO_4_.7H_2_O (35 mg, 0.125 mmol) and 1,10-phenanthroline monohydrate (25 mg, 0.125 mmol) in a total volume of 60 ml. The pH value was adjusted to 10.8 using aqueous NaOH solution and dioxygen was bubbled in for 48 h; final pH was 10.3. Initially a small amount of yellow-white precipitate came out and the reaction mixture ultimately assumed a reddish-pink tinge. It was transferred to a 100 ml beaker, requisite quantity of water was added to make up for the evaporation loss and allowed to stand at room temperature. Pink crystals suitable for single-crystal X-ray diffraction appeared after 15 days (yield: 30%).

### Refinement

The H atoms were all located in a difference map, but those attached to C atoms were repositioned geometrically. The H atoms were initially refined with soft restrains on the bond lengths and angles to regularize their geometry (C—H = 0.93–0.98, N—H = 0.86–0.89, O—H = 0.82 Å) and with *U*_iso_(H) = 1.2–1.5*U*_eq_(parent atom), after which the positions were refined with rigiding constrains.

### Crystal data

**Table:**

[Co(C_8_H_5_N_5_O_3_)(C_12_H_8_N_2_)(H_2_O)]·3H_2_O	*Z* = 2
*M**_r_* = 530.36	*F*(000) = 546
Triclinic, *P*1	*D*_x_ = 1.659 Mg m^−^^3^
Hall symbol: -P 1	Mo *K*α radiation, λ = 0.71073 Å
*a* = 8.454 (2) Å	Cell parameters from 8945 reflections
*b* = 9.934 (3) Å	θ = 2–28°
*c* = 13.778 (4) Å	µ = 0.87 mm^−^^1^
α = 97.534 (4)°	*T* = 110 K
β = 95.281 (4)°	Block, pink
γ = 110.603 (4)°	0.23 × 0.11 × 0.04 mm
*V* = 1061.8 (5) Å^3^	

### Data collection

**Table:**

Bruker Kappa APEXII CCD diffractometer	4360 reflections with *I* > 2σ(*I*)
Graphite monochromator	*R*_int_ = 0.030
φ and ω scans	θ_max_ = 28.2°, θ_min_ = 1.5°
Absorption correction: multi-scan (*SADABS*; Sheldrick, 1996)	*h* = −11→11
*T*_min_ = 0.82, *T*_max_ = 0.97	*k* = −12→13
8945 measured reflections	*l* = −18→18
4726 independent reflections	

### Refinement

**Table:**

Refinement on *F*^2^	Primary atom site location: structure-invariant direct methods
Least-squares matrix: full	Hydrogen site location: difference Fourier map
*R*[*F*^2^ > 2σ(*F*^2^)] = 0.057	H-atom parameters constrained
*wR*(*F*^2^) = 0.129	Method = Modified Sheldrick *w* = 1/[σ^2^(*F*^2^) + ( 0.04*P*)^2^ + 3.34*P*], where *P* = (max(*F*_o_^2^,0) + 2*F*_c_^2^)/3
*S* = 1.03	(Δ/σ)_max_ = 0.0001859
4726 reflections	Δρ_max_ = 0.99 e Å^−^^3^
316 parameters	Δρ_min_ = −0.88 e Å^−^^3^
0 restraints	

### Fractional atomic coordinates and isotropic or equivalent isotropic displacement parameters (Å^2^)

**Table:**

	*x*	*y*	*z*	*U*_iso_*/*U*_eq_	
Co1	0.45982 (5)	0.22172 (4)	0.22887 (3)	0.0125	
O1	0.2062 (3)	0.0747 (2)	0.23341 (17)	0.0176	
C13	0.1224 (4)	0.1182 (3)	0.2948 (2)	0.0159	
O2	−0.0205 (3)	0.0408 (2)	0.31159 (18)	0.0204	
C14	0.2096 (4)	0.2762 (3)	0.3463 (2)	0.0150	
N3	0.3618 (3)	0.3367 (3)	0.32052 (19)	0.0137	
C19	0.4572 (4)	0.4746 (3)	0.3559 (2)	0.0137	
C16	0.4012 (4)	0.5628 (3)	0.4205 (2)	0.0151	
N5	0.4986 (3)	0.7057 (3)	0.4529 (2)	0.0154	
C17	0.6493 (4)	0.7539 (3)	0.4170 (2)	0.0157	
N4	0.7169 (3)	0.6739 (3)	0.3559 (2)	0.0161	
C18	0.6243 (4)	0.5321 (3)	0.3254 (2)	0.0148	
O3	0.6704 (3)	0.4463 (2)	0.26886 (17)	0.0174	
N7	0.7460 (4)	0.8957 (3)	0.4440 (2)	0.0199	
H141	0.8293	0.9343	0.4135	0.0223*	
H142	0.7086	0.9522	0.4775	0.0228*	
N6	0.2466 (3)	0.5028 (3)	0.4504 (2)	0.0176	
C15	0.1508 (4)	0.3621 (3)	0.4146 (2)	0.0171	
C20	−0.0163 (4)	0.2992 (4)	0.4506 (3)	0.0256	
H172	−0.0359	0.3696	0.4963	0.0378*	
H173	−0.0185	0.2188	0.4829	0.0383*	
H171	−0.1061	0.2680	0.3985	0.0380*	
O4	0.5538 (3)	0.1469 (2)	0.35063 (17)	0.0185	
H181	0.4964	0.0663	0.3597	0.0272*	
H182	0.5418	0.1894	0.4013	0.0271*	
N2	0.3758 (3)	0.2801 (3)	0.0963 (2)	0.0162	
C12	0.2567 (4)	0.3370 (4)	0.0798 (3)	0.0196	
C11	0.2191 (4)	0.3750 (4)	−0.0116 (3)	0.0230	
C10	0.3071 (4)	0.3548 (4)	−0.0867 (3)	0.0220	
C8	0.4354 (4)	0.2958 (4)	−0.0719 (2)	0.0183	
C9	0.4634 (4)	0.2593 (3)	0.0218 (2)	0.0138	
C5	0.5897 (4)	0.1963 (3)	0.0422 (2)	0.0147	
N1	0.6075 (3)	0.1592 (3)	0.1330 (2)	0.0152	
C1	0.7247 (4)	0.1018 (3)	0.1537 (2)	0.0178	
C2	0.8260 (4)	0.0749 (4)	0.0839 (3)	0.0225	
C3	0.8069 (4)	0.1096 (4)	−0.0079 (3)	0.0221	
C4	0.6854 (4)	0.1721 (3)	−0.0323 (2)	0.0179	
C6	0.6545 (4)	0.2115 (4)	−0.1271 (3)	0.0227	
C7	0.5346 (5)	0.2690 (4)	−0.1461 (3)	0.0241	
H321	0.5124	0.2898	−0.2083	0.0280*	
H311	0.7136	0.1926	−0.1771	0.0268*	
H291	0.8704	0.0898	−0.0554	0.0258*	
H281	0.9086	0.0377	0.1020	0.0257*	
H271	0.7401	0.0814	0.2171	0.0208*	
H221	0.2815	0.3779	−0.1477	0.0263*	
H211	0.1346	0.4115	−0.0211	0.0270*	
H201	0.1976	0.3531	0.1304	0.0229*	
O7	0.9931 (4)	0.4695 (3)	0.1919 (3)	0.0445	
H331	1.0355	0.5568	0.1993	0.0644*	
H332	0.9309	0.4819	0.2305	0.0648*	
O5	0.0341 (3)	−0.2327 (3)	0.28207 (18)	0.0224	
H341	0.0418	−0.1559	0.2637	0.0322*	
H342	−0.0472	−0.2571	0.3124	0.0321*	
O6	0.3374 (3)	−0.0951 (2)	0.40693 (18)	0.0204	
H351	0.2468	−0.1420	0.3696	0.0287*	
H352	0.3795	−0.1552	0.4182	0.0294*	

### Atomic displacement parameters (Å^2^)

**Table:**

	*U*^11^	*U*^22^	*U*^33^	*U*^12^	*U*^13^	*U*^23^
Co1	0.0135 (2)	0.0131 (2)	0.0129 (2)	0.00622 (16)	0.00458 (15)	0.00324 (15)
O1	0.0164 (11)	0.0142 (11)	0.0210 (12)	0.0040 (9)	0.0048 (9)	0.0026 (9)
C13	0.0156 (15)	0.0157 (15)	0.0168 (15)	0.0064 (12)	−0.0002 (12)	0.0050 (12)
O2	0.0143 (11)	0.0173 (11)	0.0269 (13)	0.0015 (9)	0.0059 (9)	0.0056 (10)
C14	0.0134 (14)	0.0150 (15)	0.0185 (15)	0.0062 (12)	0.0048 (12)	0.0053 (12)
N3	0.0134 (12)	0.0130 (12)	0.0153 (13)	0.0049 (10)	0.0035 (10)	0.0038 (10)
C19	0.0139 (14)	0.0141 (14)	0.0156 (15)	0.0062 (12)	0.0053 (12)	0.0057 (12)
C16	0.0158 (15)	0.0172 (15)	0.0152 (15)	0.0085 (12)	0.0029 (12)	0.0050 (12)
N5	0.0149 (13)	0.0129 (12)	0.0196 (14)	0.0060 (10)	0.0040 (10)	0.0030 (10)
C17	0.0157 (15)	0.0175 (15)	0.0167 (15)	0.0083 (12)	0.0030 (12)	0.0063 (12)
N4	0.0150 (13)	0.0148 (13)	0.0202 (14)	0.0057 (10)	0.0078 (11)	0.0047 (11)
C18	0.0144 (15)	0.0169 (15)	0.0150 (15)	0.0065 (12)	0.0036 (12)	0.0063 (12)
O3	0.0173 (11)	0.0170 (11)	0.0193 (12)	0.0073 (9)	0.0065 (9)	0.0028 (9)
N7	0.0188 (14)	0.0136 (13)	0.0264 (15)	0.0044 (11)	0.0081 (12)	0.0020 (11)
N6	0.0164 (13)	0.0169 (13)	0.0224 (14)	0.0083 (11)	0.0071 (11)	0.0041 (11)
C15	0.0148 (15)	0.0171 (15)	0.0226 (16)	0.0075 (12)	0.0065 (12)	0.0079 (13)
C20	0.0163 (16)	0.0207 (17)	0.040 (2)	0.0056 (14)	0.0126 (15)	0.0024 (15)
O4	0.0198 (12)	0.0193 (11)	0.0174 (11)	0.0069 (9)	0.0052 (9)	0.0063 (9)
N2	0.0151 (13)	0.0150 (13)	0.0203 (14)	0.0061 (10)	0.0063 (11)	0.0055 (11)
C12	0.0169 (16)	0.0171 (15)	0.0263 (18)	0.0064 (13)	0.0075 (13)	0.0055 (13)
C11	0.0193 (17)	0.0195 (16)	0.0319 (19)	0.0082 (14)	0.0003 (14)	0.0098 (14)
C10	0.0202 (17)	0.0232 (17)	0.0224 (17)	0.0061 (14)	−0.0007 (13)	0.0107 (14)
C8	0.0177 (16)	0.0168 (15)	0.0178 (16)	0.0030 (12)	0.0007 (12)	0.0044 (13)
C9	0.0133 (14)	0.0114 (14)	0.0153 (15)	0.0026 (11)	0.0032 (11)	0.0022 (11)
C5	0.0129 (14)	0.0113 (14)	0.0176 (15)	0.0020 (11)	0.0022 (12)	0.0015 (12)
N1	0.0152 (13)	0.0133 (12)	0.0158 (13)	0.0040 (10)	0.0034 (10)	0.0013 (10)
C1	0.0171 (15)	0.0150 (15)	0.0199 (16)	0.0058 (12)	0.0002 (12)	0.0005 (12)
C2	0.0169 (16)	0.0214 (17)	0.0312 (19)	0.0103 (14)	0.0035 (14)	0.0025 (14)
C3	0.0162 (16)	0.0190 (16)	0.0298 (19)	0.0059 (13)	0.0079 (14)	−0.0016 (14)
C4	0.0152 (15)	0.0162 (15)	0.0200 (16)	0.0032 (12)	0.0055 (13)	0.0009 (13)
C6	0.0241 (17)	0.0251 (17)	0.0181 (17)	0.0072 (14)	0.0093 (14)	0.0026 (14)
C7	0.0299 (19)	0.0254 (18)	0.0169 (16)	0.0070 (15)	0.0085 (14)	0.0086 (14)
O7	0.0352 (16)	0.0272 (15)	0.074 (2)	0.0127 (13)	0.0292 (16)	−0.0010 (15)
O5	0.0178 (11)	0.0184 (12)	0.0318 (14)	0.0057 (9)	0.0095 (10)	0.0059 (10)
O6	0.0192 (12)	0.0166 (11)	0.0266 (13)	0.0077 (9)	0.0020 (10)	0.0060 (10)

### Geometric parameters (Å, º)

**Table:**

Co1—O1	2.140 (2)	N2—C12	1.333 (4)
Co1—N3	2.016 (3)	N2—C9	1.355 (4)
Co1—O3	2.270 (2)	C12—C11	1.402 (5)
Co1—O4	2.120 (2)	C12—H201	0.923
Co1—N2	2.123 (3)	C11—C10	1.363 (5)
Co1—N1	2.079 (3)	C11—H211	0.914
O1—C13	1.279 (4)	C10—C8	1.414 (5)
C13—O2	1.244 (4)	C10—H221	0.926
C13—C14	1.519 (4)	C8—C9	1.408 (4)
C14—N3	1.319 (4)	C8—C7	1.435 (5)
C14—C15	1.426 (4)	C9—C5	1.439 (4)
N3—C19	1.319 (4)	C5—N1	1.359 (4)
C19—C16	1.397 (4)	C5—C4	1.411 (4)
C19—C18	1.450 (4)	N1—C1	1.333 (4)
C16—N5	1.354 (4)	C1—C2	1.406 (5)
C16—N6	1.360 (4)	C1—H271	0.930
N5—C17	1.360 (4)	C2—C3	1.363 (5)
C17—N4	1.378 (4)	C2—H281	0.928
C17—N7	1.337 (4)	C3—C4	1.412 (5)
N4—C18	1.335 (4)	C3—H291	0.928
C18—O3	1.265 (4)	C4—C6	1.439 (5)
N7—H141	0.852	C6—C7	1.349 (5)
N7—H142	0.843	C6—H311	0.925
N6—C15	1.342 (4)	C7—H321	0.926
C15—C20	1.491 (4)	O7—H331	0.800
C20—H172	0.947	O7—H332	0.810
C20—H173	0.960	O5—H341	0.811
C20—H171	0.930	O5—H342	0.820
O4—H181	0.810	O6—H351	0.830
O4—H182	0.801	O6—H352	0.820
			
O1—Co1—N3	75.10 (10)	H172—C20—H171	106.6
O1—Co1—O3	151.22 (8)	H173—C20—H171	109.7
N3—Co1—O3	76.26 (9)	Co1—O4—H181	116.6
O1—Co1—O4	90.13 (9)	Co1—O4—H182	109.7
N3—Co1—O4	90.23 (10)	H181—O4—H182	95.0
O3—Co1—O4	92.74 (9)	Co1—N2—C12	128.8 (2)
O1—Co1—N2	90.99 (10)	Co1—N2—C9	112.7 (2)
N3—Co1—N2	96.45 (10)	C12—N2—C9	118.5 (3)
O3—Co1—N2	89.46 (9)	N2—C12—C11	122.3 (3)
O4—Co1—N2	173.29 (10)	N2—C12—H201	119.1
O1—Co1—N1	119.55 (10)	C11—C12—H201	118.6
N3—Co1—N1	164.48 (10)	C12—C11—C10	119.6 (3)
O3—Co1—N1	88.76 (9)	C12—C11—H211	120.2
O4—Co1—N1	94.58 (10)	C10—C11—H211	120.2
N2—Co1—N1	79.12 (10)	C11—C10—C8	119.9 (3)
Co1—O1—C13	116.8 (2)	C11—C10—H221	120.1
O1—C13—O2	124.1 (3)	C8—C10—H221	120.0
O1—C13—C14	114.6 (3)	C10—C8—C9	116.7 (3)
O2—C13—C14	121.2 (3)	C10—C8—C7	124.4 (3)
C13—C14—N3	111.4 (3)	C9—C8—C7	118.9 (3)
C13—C14—C15	129.9 (3)	C8—C9—N2	123.1 (3)
N3—C14—C15	118.8 (3)	C8—C9—C5	120.1 (3)
Co1—N3—C14	121.6 (2)	N2—C9—C5	116.8 (3)
Co1—N3—C19	117.6 (2)	C9—C5—N1	117.5 (3)
C14—N3—C19	120.8 (3)	C9—C5—C4	119.5 (3)
N3—C19—C16	121.8 (3)	N1—C5—C4	123.0 (3)
N3—C19—C18	117.4 (3)	Co1—N1—C5	113.6 (2)
C16—C19—C18	120.7 (3)	Co1—N1—C1	127.6 (2)
C19—C16—N5	120.8 (3)	C5—N1—C1	118.5 (3)
C19—C16—N6	118.7 (3)	N1—C1—C2	122.0 (3)
N5—C16—N6	120.4 (3)	N1—C1—H271	118.0
C16—N5—C17	115.1 (3)	C2—C1—H271	120.0
N5—C17—N4	127.9 (3)	C1—C2—C3	119.8 (3)
N5—C17—N7	117.0 (3)	C1—C2—H281	119.3
N4—C17—N7	115.1 (3)	C3—C2—H281	120.9
C17—N4—C18	117.6 (3)	C2—C3—C4	119.9 (3)
C19—C18—N4	117.7 (3)	C2—C3—H291	120.7
C19—C18—O3	118.1 (3)	C4—C3—H291	119.4
N4—C18—O3	124.2 (3)	C3—C4—C5	116.8 (3)
Co1—O3—C18	110.63 (19)	C3—C4—C6	124.2 (3)
C17—N7—H141	119.8	C5—C4—C6	119.0 (3)
C17—N7—H142	119.9	C4—C6—C7	121.2 (3)
H141—N7—H142	117.6	C4—C6—H311	119.5
C16—N6—C15	119.0 (3)	C7—C6—H311	119.2
C14—C15—N6	120.8 (3)	C8—C7—C6	121.3 (3)
C14—C15—C20	121.7 (3)	C8—C7—H321	118.4
N6—C15—C20	117.4 (3)	C6—C7—H321	120.3
C15—C20—H172	111.5	H331—O7—H332	86.2
C15—C20—H173	110.1	H341—O5—H342	108.7
H172—C20—H173	108.2	H351—O6—H352	105.5
C15—C20—H171	110.7		

### Hydrogen-bond geometry (Å, º)

**Table:**

*D*—H···*A*	*D*—H	H···*A*	*D*···*A*	*D*—H···*A*
N7—H141···O2^i^	0.85	2.12	2.942 (4)	163
N7—H142···O6^ii^	0.84	2.15	2.970 (4)	165
O4—H181···O6	0.81	1.93	2.717 (3)	164
O4—H182···N5^ii^	0.80	2.25	3.051 (4)	176
O5—H341···O1	0.82	2.34	3.079 (4)	151
O5—H341···O2	0.82	2.23	2.896 (4)	139
O5—H342···N4^iii^	0.82	2.04	2.844 (4)	166
O6—H351···O5	0.83	1.92	2.740 (4)	174
O6—H352···N5^iv^	0.82	2.05	2.871 (4)	176
O7—H331···O5^i^	0.80	2.25	2.941 (4)	145
O7—H332···O3	0.81	2.23	2.962 (5)	151

Symmetry codes: (i) *x*+1, *y*+1, *z*; (ii) −*x*+1, −*y*+1, −*z*+1; (iii) *x*−1, *y*−1, *z*; (iv) *x*, *y*−1, *z*.
